# Cross-cultural adaptation and validation of a questionnaire on eating habits and physical activity of university students in confinement due to coronavirus disease

**DOI:** 10.1017/S1368980022000805

**Published:** 2022-03-31

**Authors:** Sebastian Cofre, Victoria Perez, Nicole Giuras, Jose L Pino, Claudio Diaz, Graciela Arguello

**Affiliations:** 1 Escuela de Nutrición y Dietética, Facultad de Ciencias de la Salud, Universidad Católica Del Maule, Talca, Chile; 2 Escuela de Nutrición y Dietética, Facultad de Salud, Universidad Santo Tomás, Chile; 3 Escuela de Trabajo Social, Facultad de Ciencias Sociales y Económicas, Universidad Católica Del Maule, Talca, Chile; 4 Department of Health Sciences, Nutrition and Dietetics Career, Faculty of Medicine, Pontificia, Universidad Católica de Chile, Santiago, Chile

**Keywords:** COVID-19, Coronavirus, Eating habits, Lifestyle, Quarantine, Validity tool

## Abstract

**Objective:**

The purpose of the current study was to cross-culturally adapt and validate an online questionnaire to assess eating habits and physical activity of university students under confinement due to coronavirus disease (COVID-19).

**Design:**

Generation of a cross-sectional online survey to university students conducted during confinement due to COVID-19. The study was divided into two phases.

**Settings:**

Students, Chile.

**Participants:**

Phase 1 considered the process of translation and back translation, expert panel, cultural adaptation and the generation of a pilot to validate a preliminary format of the questionnaire. In Phase 2, information from the instrument was collected from two hundred and sixty-eight university students, ages 16 to 30 years old, with a mean age of 21·6 (3·3) The major proportion of participants were female (82 %).

**Results:**

The adapted questionnaire was statistically validated in three dimensions: (A) eating habits and behaviours during quarantine, (B) perception of risk and (C) physical activity changes during the quarantine. The reliability of Cronbach’s *α* for dimensions A, B and C was 0·59, 0·85 and 0·97, respectively. The complete questionnaire obtained 0·61 in internal consistency and 0·61 (0·58–0·67) ICC reliability. A statistically significant positive correlation matrix was observed.

**Conclusions:**

This questionnaire is a practical tool to obtain accurate information about the relation of COVID-19 confinement on people’s eating habits and physical activity. Therefore, it could contribute to establishing appropriate strategies to prevent negative effects on people’s health.

The unprecedented coronavirus disease (COVID-19) pandemic has required health organisms, public health experts and government officials to proceeds several measures to contain the spread of infections and prevent the massive death of the affected population. They have accomplished this by setting up strict quarantines and strengthening health facilities to control the disease until effective vaccines are available^(1)^. In several countries, including Chile, the effort to reduce the spread of the COVID-19 virus (SARS-CoV-2) among the younger and adult populations has provoked the extensive closure of schools, colleges, universities and other educational institutions^(2)^.

However, although extremely necessary, the first evidence of the confinement impacts shows an increase in people’s stress and anxiety^(3)^, particularly in college students^(4)^. Likewise, worsened people’s eating habits and lifestyles have also been observed^(5–7)^. So, it is likely that unhealthy habits like higher consumption of processed foods with higher energetic content, saturated fats, sugars and refined carbohydrates and easier access^(8)^, in addition to physical inactivity, anxiety and stress caused by this uncertain and confined scenario, can promote to increasing the prevalence of obesity in the times of COVID-19^(9)^.

On the other hand, adequate dietary intake and healthy lifestyles may be essential to safeguard against an excessive inflammatory response to SARS-Cov-2 infection, preventing the progress of the infection to severe or improving its result^(10)^. As recently debated by the European Society for Clinical Nutrition and Metabolism^(11)^, the obesity condition is hazardous to the severity of COVID-19. Obesity has emerged as one of the most well-known risk factors increasing disease mortality^(12,13)^. In this sense, nutritional status, eating habits and physical activity might influence the individual risk for the progression of SARS-CoV-2; however, information is incipient.

To know the potential impact of the COVID-19 outbreak on the eating habits and lifestyles, validated instruments culturally adapted to the local reality^(14)^ of each country are needed. This will lead to information for public health decision making and establish appropriate strategies in future re-emerging outbreaks. Therefore, the current study is aimed to carry out cross-cultural adaptation and validate an English-to-Spanish questionnaire on the eating habits and physical activity of university students under confinement due to COVID-19 pandemic.

## Methods

### Study design

The present cross-sectional online study was developed in two phases: phase 1 considered the process of translation and back translation, expert panel, cultural adaptation and the generation of a pilot to validate a preliminary format of the questionnaire and the phase 2 consisted in the verification of the validity trough determining the metric properties of the scale.

### Phase 1: cross-cultural adaptation questionnaire

The original questionnaire called ‘Dietary choices and habits during COVID-19 Lockdown’ was developed by Sidor *et al.*^(15)^ in Poland in the English language. The method of translation was performed by ten bilingual dietitians committee, who translated the original English version of the questionnaire to a Spanish version. This committee compared the consistency and the adaptation of the questions and answers. The conceptual equivalence was evaluated with a Likert scale from 1 to 7, where 1 was ‘Very understandable/Very equivalent’ and 7 was “Poorly understandable/not equivalent considered the participation. Subsequently, two independent evaluators carried out the back translation of the instrument to identify possible errors in the items of the preliminary questionnaire. Another committee, composed of four experts from the area of eating behaviour, critically analysed the resulting instrument, verifying the cultural relevance and construct validity for the target population.

**Fig. 1:**
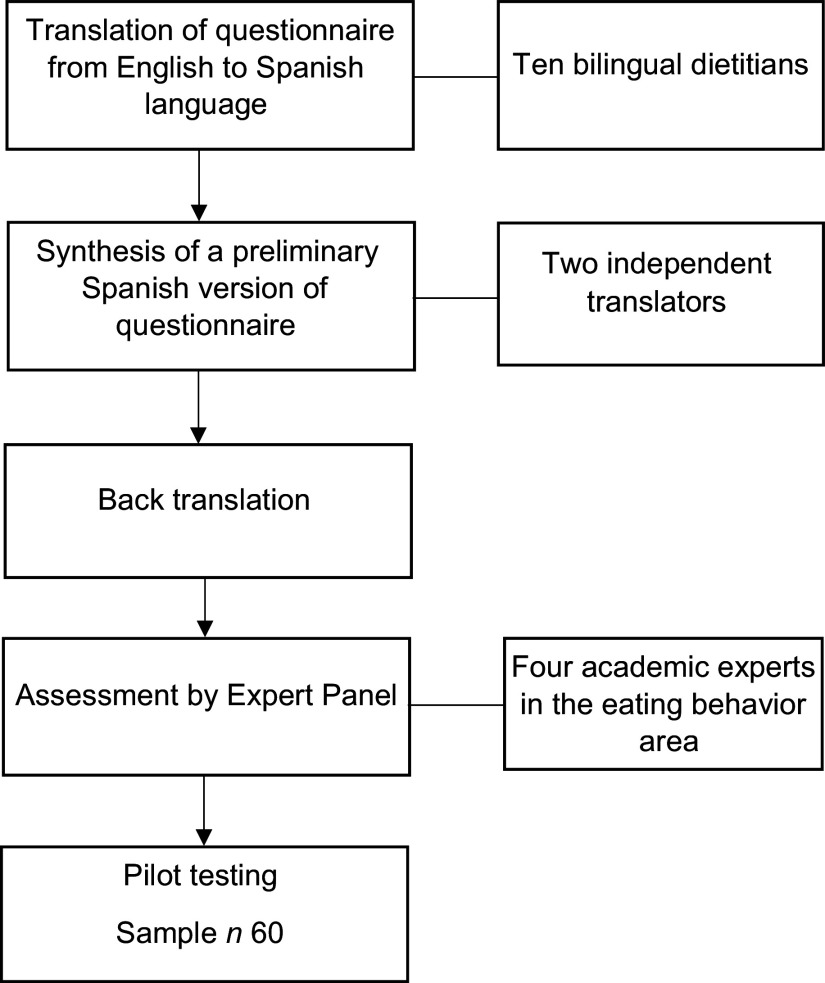
The process of cross-cultural adaptation

Afterward, a pilot study of the online questionnaire was carried out through the Google Form platform. Sixty university students from the Faculty of Health Sciences evaluated face validity, cultural adaptation and instrument relevance. The students also verified the feasibility of applicability and comprehension of the text. Finally, with the data collected and the observations made, the instrument’s final version was adjusted and applied. This process is presented in Fig. 1.

### Phase 2: reliability and validity of the questionnaire

It is important to highlight that the original questionnaire does not declare a reliability analysis. The questionnaire adapted to the Spanish language included two parts: (1) questions related with the symptoms, contact and hospitalisation for COVID-19 and (2) Questionnaire composed of three dimensions on eating habits, fear of contact with food and physical activity during confinement.

The part 1 relationship was not subjected to consistency analysis, since it only relates to the absence or presence of symptoms, signs or hospitalisation related to COVID-19.

The procedure of the reliability evaluation of part 2 is presented below:

#### (A) Eating habits and behaviours during quarantine

In total, nineteen answers reported the eating habits and conduct during the quarantine due to COVID-19. A daily number of meals and snacks consumed, cooking practices, the frequency of consumption of selected food products (fruits, fresh vegetables, legumes, grains, meat products, dairy products, fast foods, processed foods, sweets and salty snacks) and the frequency of breakfast consumption. The responses were rated on a Likert scale.

#### (B) Perception of risk

This dimension considered two questions about the identification of the level of fear of getting infected by SARS-CoV-2 during grocery shopping and through contact with food products. The responses were rated on a Likert scale.

#### (C) Physical activity

Two questions that indicate the level of physical activity development during quarantine: (i) please indicate if you have done any physical activity during the quarantine and (ii) if you have been physically active during quarantine, please indicate how often you have done. The responses were rated on a Likert scale.

The link to the instrument was sent by mass email. The data were conducted through the Google Form platform for 3 weeks from 24 August 2020 to 11 September 2020. The data collection process was anonymous to guarantee the participants’ health safety in pandemic conditions.

### Sample

To evaluate the metric properties (reliability and validity) of the Spanish version of the questionnaire, the sample size was calculated based on the recommendations of Streiner *et al.*^(16)^ An instrument was applied whose sample was non-probabilistic for convenience, since it is a sample that allows the selection of those accessible cases that agree to be included^(17)^. This fundamental in the convenient accessibility and proximity of the subjects. In our case, the information gathering strategy turned out to be relevant because the instrument disseminated by e-mail and social networks gave us an amount of sample defined by the voluntary nature of its application. The eligibility criteria applied were students enrolled in the four university faculties: Health of Sciences, Education, Medicine and Agricultural and Forestry Sciences. These four faculties gave their explicit intention to participate in the current study. Finally, the questionnaire was applied, and the feedback was consolidated, analysed and discussed by the research team.

### Statistical analysis

Descriptive statistics were applied to categorical variables and expressed as frequencies and percentages. The quantitative variables were expressed as means and standard deviations.

For the reliability of agreement in translation and back translation was calculated Fleiss’ *κ* and Cohen’s *κ*, respectively. The reliability was analysed following two approaches: the internal consistency was evaluated using Cronbach’s *α*, and intraobserver reliability was considered the agreement of the values between the cases themselves was established with the intraclass correlation coefficient (ICC) with a mixed random model of two factors for a consistency-type index for each dimension. As a rule of thumb, the coefficient for Cronbach’s *α* was interpreted following George and Mallery criteria, that is to say, <0·5 ‘Unacceptable’; >0·5 ‘Poor’; >0·6 ‘Moderate’; >0·7 ‘Acceptable’; >0·8 ‘Good’; >0·9 ‘Excellent’^(18)^. For the ICC was considered, values < 0·5 are indicative of ‘Poor reliability’, values between 0·5 and 0·75 indicate ‘Moderate reliability’, values between 0·75 and 0·9 indicate ‘Good reliability’ and values greater than 0·90 indicate ‘Excellent reliability’^(19)^. *P* values <0 05 were considered as significant. The data analyses were performed using SPSS Statistics for Windows, Version 19.0 (IBM Corp.).

## Results

### Cross-cultural adaptation process

The results of the translation of the questionnaire are presented in Table 1. The mean of interpretability and conceptual equivalence for the dimensions A, B and C were 1·97, 1·20 and 1·0, respectively. The statistic Fleiss *κ* was 0·86 between the evaluators.

**Table 1:** Results of average interpretability and conceptual equivalence reported by ten bilingual dietitians in the translation process

Dimensions	Questions	Mean	sd	Mean/dimension
A	Q1	2·00	1·86	1·97
	Q2	1·75	1·06	
	Q3	2·33	1·83	
	Q4	2·00	1·86	
	Q5	2·00	1·86	
	Q6	2·50	2·11	
	Q7	1·75	1·06	
	Q8	1·92	1·62	
	Q9	2·25	1·82	
	Q10	1·58	1·51	
	Q11	1·83	1·47	
	Q12	2·00	1·48	
	Q13	2·00	1·60	
	Q14	1·83	1·47	
	Q15	3·42	2·39	
	Q16	1·50	1·17	
	Q17	1·25	0·87	
	Q18	1·00	0·00	
	Q19	1·00	0·00	
	Q20	1·58	1·00	
B	Q21	1·33	0·65	1·2
	Q22	1·08	0·29	
C	Q23	1·00	0·00	1·0
	Q24	1·00	0·00	

In the back translation process, a concordance agreement was applied where 92 % was obtained among the evaluators. In the case of *κ* Cohen´s obtained was 0·77. In the pilot study, the students showed an adequate understanding and face validity of the instruments, highlighting being friendly and understandable to answer.

### Demographic characteristics

Two hundred and sixty-eight university students participated in the validation of the instrument according to eligibility criteria. The summary of demographic characteristics is presented in Table 2. A mean age of 21·6 (3·3) years was obtained, observing greater participation in women (82 %). According to their geographical distribution, there is a higher frequency of participants from the country’s middle area, representing 94·7 %. Concerning their membership to the university faculties, a higher percentage of students that participated are from the Faculty of Health Sciences (51·1 %), followed by Education (26·8 %), Medicine (14·5 %) and the Faculty of Agricultural and Forestry Sciences (7·4 %).

**Table 2:** Demographic breakdown of surveyed participants

Variables	Female *n* 220		Male *n* 48		Overall *n* 268	
	*n*	%	*n*	%	*n*	%
Age in years						
Mean	21·6		21·7		21·6	
sd	3·3		3·4		3·3	
District						
North	7	2·6	1	0·4	8	3·0
Middle	208	77·6	46	17·1	254	94·7
South	5	1·9	1	0·4	6	2·2
Faculty						
Health of Sciences	120	44·7	17	6·3	137	51·1
Education	57	21·2	15	5·6	72	26·8
Medicine	29	10·8	10	3·7	39	14·5
Agricultural and Forestry Sciences	14	5·2	6	2·2	20	7·4
Weeks in quarantine	21·7	22·0	18·0	11·0	21·1	20·5
Self-report						
Bodyweight, kg						
Mean	65·8		76·0		67·6	
sd	14·0		13·1		14·4	
Size, cm						
Mean	159·6		169·3		161·4	
sd	9·0		12·2		10·3	
BMI, kg/m^2^						
Mean	25·9		26·9		26·1	
sd	5·4		6·1		5·6	
Nutritional status						
Normal	113	42·1	23	8·5	136	50·6
Overweight	64	23·8	14	5·2	78	29·0
Obesity	39	14·5	10	3·7	49	18·2

Values are presented as mean and sd or numbers and percentages.

Regarding the time of quarantine, a mean of 21·1 (20·5) weeks was observed, with the mean confinement being higher in the group of women (21·7 weeks).

The self-report of body weight was higher in men with 76·0 (13·1) g compared with women with 65·8 (14·0) kg. It was observed greater malnutrition was reported in the female group (overweight 23·8 % and obesity 14·5 %). It is important to note that according to the self-report, 50 % of the students possess a normal nutritional status according to the BMI.

### Psychometric properties

Table 3 shows the analysis of the internal consistency or reliability of the questionnaire.

**Table 3:** Internal consistency of the questionnaire

Domain	Questions	Mean	sd	Corrected item-total correlation	Cronbach *α* if item deleted	Cronbach *α*	Intraclass correlation (CI)	
Overall		3·03	1·24			0·61	0·61	0·58–0·67†
Dimension A						0·59	0·59	0·52–0·66†
	Q1	3·51	1·13	0·34	0·56			
	Q2	4·24	0·99	0·35	0·57			
	Q3	3·43	1·12	0·36	0·56			
	Q4	1·61	0·95	0·22	0·58			
	Q5	3·09	1·27	0·12	0·59			
	Q6	4·03	1·05	0·11	0·59			
	Q7	4·15	1·68	0·08	0·60			
	Q8	5·05	1·30	0·17	0·58			
	Q9	3·55	0·98	0·14	0·59			
	Q10	3·53	1·75	0·16	0·59			
	Q11	3·31	1·48	0·24	0·57			
	Q12	3·00	1·39	0·20	0·58			
	Q13	4·26	1·83	0·24	0·57			
	Q14	2·41	1·31	0·24	0·57			
	Q15	2·40	1·42	0·21	0·58			
	Q16	3·05	1·84	0·20	0·58			
	Q17	2·49	1·57	0·21	0·58			
	Q18	2·66	1·51	0·25	0·57			
	Q19	2·49	1·74	0·13	0·59			
	Q20	3·30	1·97	0·20	0·58			
Dimension B	Q21	3·44	1·44	0·27	–	0·85	0·73	0·67–0·78†
	Q22	2·89	1·50	0·37	–			
Dimension C	Q23	2·67	1·19	0·19	–	0·97	0·94	0·92–0·95†
	Q24	2·54	1·13	0·19	–			

† *P* < 0·001.

Dimension A presented a Cronbach’s *α* of 0·59. No questions were excluded from this analysis. Alpha’s value raised to 0·60 by eliminating the question oriented to fruit consumption (Q7), but its elimination is not justified given the nutritional importance of the question. The intraclass correlation was 0·59 (0·52–0·66).

In the case of dimension B (Q21–Q22), related with the perception of risk when buying food, showed a Cronbach’s *α* of 0·85. and ICC 0·73 (0·67–0·78).

Finally, dimension C (Q23–Q24), related with of level of physical activity during confinement, presented a Cronbach’s *α* of 0·97 and ICC 0·94 (0·92–0·95). Eliminating any of these elements lowers the Cronbach’s *α* value below 0·5, so the instrument presented an *α* of 0·61. The part I (COVID-19 relationship) was not subjected to consistency analysis, since it only relates to the absence or presence of symptoms related to COVID-19. Part 1 of the questionnaire asked about symptoms, contact and hospitalisation for COVID-19, they were not subjected to reliability analysis because they were dichotomous questions.

A significant positive correlation was identified between the dimensions A, B and C (*r* = 0·21, *P* < 0·01), that is, the higher the perception of changes, the higher the score in the dimension eating habits and behaviours in the pandemic, as shown in Table 4. It is relevant to mention that two of the three dimensions presented high and significant correlations with the dimensions total score; however, among them the correlations were low.

**Table 4:** Correlation matrix of the questionnaire domains

Dimensions	Eating habits and behaviours in pandemic	Perception of risk	Physical activity
Eating habits and behaviours in pandemic (A)	1		
Perception of risk (B)	0·074	1	
Physical activity (C)	−0·082	0·069	1

*R* = 0·21.

## Discussion

Among the main findings observed, we found that the validated instrument seems to have adequate reliability to evaluate the eating habits and physical activity of university students during confinement due to COVID-19. Current literature analyses the relation of COVID-19 on the population; however, few studies indicate any cultural adaptation, validity and reliability of the instrument applied. The use of validated instruments culturally adapted to each country’s local reality allows obtaining sensitive, specific and reproducible data to make decisions. In this direction, applied studies in university students have shown that psychometric instruments’ validation allows an adequate evaluation of eating behaviour and lifestyle^(20,21)^. Studies related to health and eating habits in university students generally obtained an important female participation, as has been shown in the studies by Musaiger *et al.*^(22)^ (85 % female participation), Yahia *et al.*^(23)^ (73 %) and Nastaskin *et al.*^(24)^ (83 %) among others, perhaps related to the fact that women have more health problems than men, are more oriented toward prevention and health education^(25)^ and, in opposite they may be influenced by external factors when making decisions related to health, so women may be more interested in health and nutrition than men.

First studies show that COVID-19 confinement and measures of its containment have a significant impact on the population’s lifestyle-related behaviour, particularly reducing physical activity and changing eating habits^(26,27)^. This situation is critical considering the current obesity epidemic^(28,29)^.

In the current study, we translated to Spanish the English language questionnaire of Sidor *et al.*^(15)^, with no previous validation and we culturally adapted it to Chilean reality. This questionnaire demonstrated moderate reliability in university students, considering the Cronbach’s *α* of 0·61 for the first statistical validation of the instrument. Some authors suggest that in the first phases of the investigation, *α* values between 0·5 and 0·6 may be sufficient so that later other research groups test and refine the instrument^(30,31)^. So too, ICC obtained a 0·61 (0·58–0·67) reporting, in the same direction of internal consistency, a moderate reliability in intraobserver evaluation.

The present questionnaire is a short, concise and user-friendly tool possible to be completed in 10 to 15 min, avoiding respondents’ lack of patience and, consequently, inaccuracy in the assessment. It consists of three dimensions covering all critical information required to assess the participants’ eating habits (intake, meal pattern and snack consumption) and physical activity.

This study’s limitations are reporting bias due to the web-based survey, social desirable bias, memory bias and inability to determine the concurrent and predictive validity, which would require a long-term follow-up. Another attribute is that this questionnaire would only be applicable to a population with similar characteristics to those of the characterised sample^(32)^, being an important characteristic to be considered by other researchers who wish to replicate the current study. It is reinforced the potential to assess the eating habits and physical activity-related behaviour of university students during the COVID-19 pandemic in Chile and Spanish-speaking countries. Overall, the questionnaire applied in the current study was shown to be reproducible and valid.

## Conclusions

This questionnaire is a practical tool to obtain accurate information about the relation of COVID-19 confinement on people’s eating habits and physical activity. Therefore, it could contribute to establishing appropriate strategies to prevent negative effects on people’s health.

## Supplementary material

For supplementary material accompanying this paper visit https://doi.org/10.1017/S1368980022000805. click here to view supplementary material
