# Supplementary material for: Cross-cultural adaptation and validation of a questionnaire on eating habits and physical activity of university students in confinement due to coronavirus disease
Source: Public Health Nutr. 2022 Mar 31;25(10):2702–8. doi: 10.1017/S1368980022000805 (PMC9273725; doi:10.1017/S1368980022000805)
Supplement: Supplementary file 1 [file S1368980022000805sup001.pdf]

Cuestionario sobre el efecto del confinamiento sanitario por COVID-19 sobre los hábitos alimentarios y estilo de vida de estudiantes universitarios

Datos sociodemográficos
Indique su edad: ________

Sexo: a) Femenino b) Masculino

Indique la región en la que vive: ______________

Indique la comuna donde realiza/ó el confinamiento por Covid-19: _____________

Indique el año de ingreso a la Universidad _______________

Indique que carrera cursa actualmente ___________________________

Indique su peso actual (en kilogramos) _____________

Indique su talla (en centímetros) ____________

¿Cuántos días llevas en confinamiento? (incluido el día de hoy) _________________

A. Con respecto a la infección por COVID-19, durante los días de confinamiento (marque, su elección).

Ha presentado síntomas compatibles con infección por COVID-19
a) Sí b) No

Ha tenido contacto estrecho (menos de 1 metro) con alguna persona con síntomas compatibles con infección por COVID-19
a) Sí b) No

Ha convivido con personas con síntomas compatibles con infección por COVID-19
a) Sí b) No

Ha tenido un diagnóstico positivo de infección por COVID-19
a) Sí b) No

Ha sido hospitalizado debido a la infección por COVID-19
a) Sí b) No

Ha sido hospitalizado en la Unidad de Cuidado Intensivo (UCI/UTI) debido a la infección por COVID-19
a) Sí b) No

B. Hábitos alimentarios durante la cuarentena

¿Ha consumido mayor cantidad de alimentos durante la cuarentena?

a) Siempre b) La mayoría de las veces c) tal vez d) Muy pocas veces e) Nunca

Indique cuántas veces come al día durante la cuarentena:

a) Una b) Dos c) Tres d) Cuatro e) Cinco d) Seis o mas

¿Ha consumido "snacks" o "bocadillos" con más frecuencia de lo habitual durante la cuarentena?

a) Siempre b) La mayoría de las veces c) tal vez d) Muy pocas veces e) Nunca

¿Ha cocinado más de lo normal durante la cuarentena?

a) Siempre b) La mayoría de las veces c) tal vez d) Muy pocas veces e) Nunca

¿Con qué frecuencia ha desayunado durante la cuarentena?

a) Todos los días b) Casi todos los días c) A veces d) Prácticamente nunca e) Nunca

Estime la frecuencia de consumo de determinados alimentos durante la cuarentena de COVID-19:

	>1 vez al día	Una vez al día	Pocas veces por semana	Una vez por semana	Una vez por mes	Ocasionalmente	Nunca
Vegetales y frutas							
Legumbres							
Cereales (Pan, Arroz, Fideos)							
Carnes blancas							
Carnes Rojas							
Lácteos							
Comida rápida							
Azúcares							
Snacks salados							
Café							
Té							

C. Estilo de vida durante la cuarentena

¿Ha notado algún cambio en su peso corporal durante la cuarentena?

a) Aumentó b) Disminuyó c) Sin cambios d) No lo sé

En caso de evidenciar cambios en su peso corporal durante la cuarentena, por favor indique la diferencia o aumento observado (en kilogramos)

Disminución en: ___ Kg Aumento en: ____Kg

¿Fumas cigarros?

a) Sí b) No

En caso de fumar, ¿ha observado una tendencia a fumar más durante la cuarentena?

a) Siempre b) La mayoría de las veces c) tal vez d) Muy pocas veces e) Nunca

¿Ha bebido mayor cantidad de alcohol durante la cuarentena?

a) Siempre b) La mayoría de las veces c) tal vez d) Muy pocas veces e) Nunca

Por favor indique si ha sentido temor a contagiarse con coronavirus durante las compras de alimentos

a) Siempre b) La mayoría de las veces c) tal vez d) Muy pocas veces e) Nunca

Por favor indique si ha realizado alguna actividad física durante la cuarentena

a) Siempre b) La mayoría de las veces c) tal vez d) Muy pocas veces e) Nunca

Si ha realizado actividad física durante la cuarentena, por favor, indique con qué frecuencia lo ha hecho

a) Siempre b) La mayoría de las veces c) tal vez d) Muy pocas veces e) Nunca

Por favor indique si ha sentido temor a contagiarse con coronavirus durante el contacto con alimentos

a) Siempre b) La mayoría de las veces c) tal vez d) Muy pocas veces e) Nunca

Actividades ocupacionales desarrolladas durante la cuarentena. Indique cuál de las siguientes actividades (una o más) sigue realizando durante la cuarentena

__Estudio vía online __Trabajo regularmente online __Trabajo regularmente de forma presencial __Suspendí mis estudios
